# Role of Carnitine Acetyl Transferase in Regulation of Nitric Oxide Signaling in Pulmonary Arterial Endothelial Cells

**DOI:** 10.3390/ijms14010255

**Published:** 2012-12-21

**Authors:** Shruti Sharma, Xutong Sun, Saurabh Agarwal, Ruslan Rafikov, Sridevi Dasarathy, Sanjiv Kumar, Stephen M. Black

**Affiliations:** Pulmonary Vascular Disease Program, Vascular Biology Center, 1459 Laney Walker Blvd, Georgia Health Sciences University, Augusta, GA 30912, USA; E-Mails: xsun@georgiahealth.edu (X.S.); saggarwal@georgiahealth.edu (S.A.); rrafikov@georgiahealth.edu (R.R.); sdasarathy@georgiahealth.edu (S.D.); skumar@georgiahealth.edu (S.K.)

**Keywords:** carnitine acetyl transferase, superoxide dismutase, nitric oxide, peroxynitrite, endothelial nitric oxide synthase

## Abstract

Congenital heart defects with increased pulmonary blood flow (PBF) result in pulmonary endothelial dysfunction that is dependent, at least in part, on decreases in nitric oxide (NO) signaling. Utilizing a lamb model with left-to-right shunting of blood and increased PBF that mimics the human disease, we have recently shown that a disruption in carnitine homeostasis, due to a decreased carnitine acetyl transferase (CrAT) activity, correlates with decreased bioavailable NO. Thus, we undertook this study to test the hypothesis that the CrAT enzyme plays a major role in regulating NO signaling through its effect on mitochondrial function. We utilized the siRNA gene knockdown approach to mimic the effect of decreased CrAT activity in pulmonary arterial endothelial cells (PAEC). Our data indicate that silencing the CrAT gene disrupted cellular carnitine homeostasis, reduced the expression of mitochondrial superoxide dismutase-and resulted in an increase in oxidative stress within the mitochondrion. CrAT gene silencing also disrupted mitochondrial bioenergetics resulting in reduced ATP generation and decreased NO signaling secondary to a reduction in eNOS/Hsp90 interactions. Thus, this study links the disruption of carnitine homeostasis to the loss of NO signaling observed in children with CHD. Preserving carnitine homeostasis may have important clinical implications that warrant further investigation.

## 1. Introduction

Children with congenital heart defects (CHD) that result in increased pulmonary blood flow (PBF) develop early and progressive alterations in pulmonary vascular function that cause significant morbidity [1]. The mechanisms involved in this pulmonary vascular disease are not fully understood, however, endothelial injury is thought to be an early hallmark [2,3]. Compelling evidence suggests that impaired NO signaling and oxidative stress play a key role in these events [4].

Oxidative and nitrative stress occurs when generation of reactive oxygen (ROS)- or nitrogen (RNS)-species overwhelms the cells natural antioxidant defenses, resulting in cellular damage and impaired function. Four enzyme systems are thought to predominate in vascular endothelial ROS generation: NADPH oxidase, xanthine oxidase, uncoupled eNOS and mitochondrial electron leakage. Whereas, the former three have been extensively studied, the role of mitochondrial derived ROS in the vascular endothelium has received less attention [5]. Mitochondria, through oxidative phosphorylation, are considered the major source of ROS in most mammalian cells. At the same time, mitochondria are potential targets of ROS action. Thus, increased ROS can damage DNA, proteins and lipids within the mitochondria, leading to alterations in the respiratory chain resulting in decreased energy production and a further increase in ROS generation (“ROS-induced ROS release”) [6,7]. In recent years, mitochondrial dysfunction has been acknowledged as a critical event in numerous pathologic conditions associated with oxidative stress, including diabetes mellitus, chronic renal failure, and neurodegenerative or cardiovascular diseases [5,8–10], and different antioxidants are being explored as potential therapeutic tools [11–13]. However, the contribution of the mitochondria to the pathogenesis of pulmonary vascular disease has been less well studied.

Previously, we have developed a clinically relevant animal model of a CHD with increased PBF, by placing a large aorto-pulmonary vascular graft (shunt) in the late-gestation fetal lamb [14], which allows the study of early mechanisms of pulmonary vascular disease. In this model, we have shown a selective impairment of endothelium-mediated pulmonary vasodilation [15,16], associated with decreased NO signaling and increased oxidative stress [2,17,18]. We have recently found mitochondrial dysfunction in these shunt lambs which was associated with the disruption of carnitine homeostasis [19]. We also correlated the disruption of carnitine homeostasis with significant decreases in both CrAT expression and activity as well as reduced NO signaling [19]. Thus, the objective of this study was to determine if loss of CrAT enzyme activity was sufficient to disrupt carnitine homeostasis, mitochondrial function, and NO signaling using a siRNA-based approach in cultured pulmonary arterial endothelial cells (PAEC).

## 2. Results

### 2.1. CrAT siRNA Significantly Decreases CrAT Protein Levels and Disrupts Carnitine Homeostasis in Pulmonary Arterial Endothelial Cells

**Figure 1:**
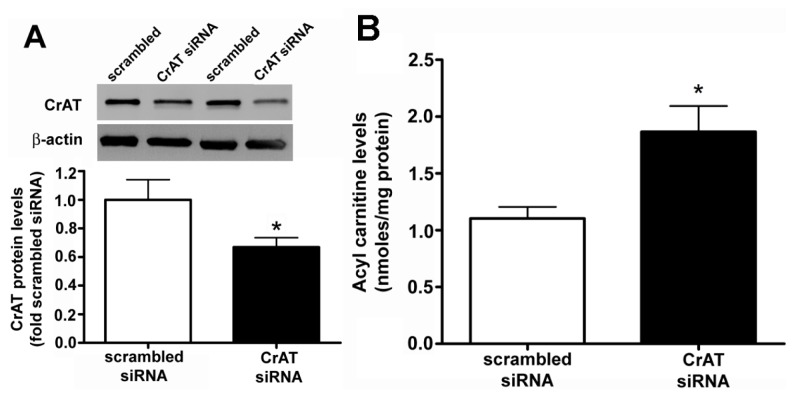
Carnitine acetyl transferase (CrAT) gene silencing decreases CrAT protein levels and disrupts carnitine homeostasis in pulmonary arterial endothelial cells. PAEC were transfected with a scrambled small interfering RNA (siRNA) or a specific CrAT siRNA for 48 h and the level of CrAT knockdown was confirmed by measuring CrAT protein levels. There was a significant reduction in CrAT protein (**A**) β-actin as used to normalize protein loading. The decrease in CrAT protein correlated with a disrupted carnitine homeostasis as demonstrated by an increase in the cellular levels of acylcarnitines (**B**) Values are mean ± SEM; *N* = 6–11. *p < 0.05 *vs.* scrambled siRNA.

PAEC were transiently transfected with a scrambled or CrAT siRNA and the level of CrAT knockdown was determined by measuring protein levels. There was a significant reduction in CrAT protein in CrAT siRNA transfected cells (Figure 1A). Carnitine is present in the form of either free carnitine or acylcarnitines. Enzymatic alterations in carnitine metabolism can result in higher levels of acylated carnitines and it is important that the potentially toxic acyl-groups are removed from the system by the administration of carnitine. To determine if the knockdown of CrAT gene resulted in disruption of cellular carnitine homeostasis, we determined acylcarnitine levels using HPLC analysis. Our data indicate that CrAT siRNA transfection led to a significant increase in acylcarnitine levels (Figure 1B), thus confirming that decreasing CrAT activity is sufficient to disrupt carnitine homeostasis in PAEC.

### 2.2. CrAT Knockdown Disrupts Superoxide Dismutase Function and Increases Mitochondrial Oxidative Stress in Pulmonary Arterial Endothelial Cells

**Figure 2:**
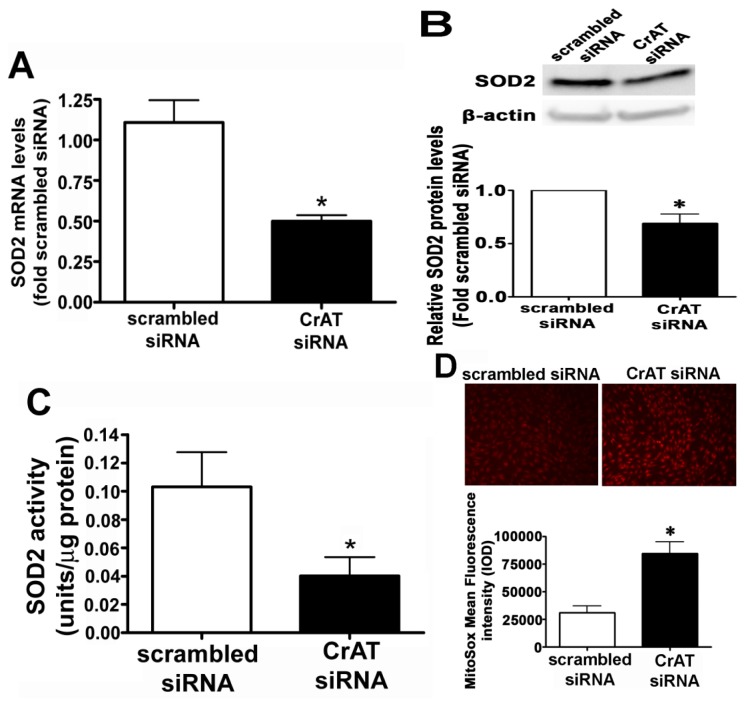
Decreased CrAT activity attenuates SOD2 expression and activity in pulmonary arterial endothelial cells. PAEC were transiently transfected with a scrambled siRNA or a specific CrAT siRNA for 48 h. Decreasing CrAT expression resulted in significant decreases in (**A**) SOD2 mRNA; (**B**) protein and (**C**) SOD2 activity. The decrease in SOD2 activity also significantly increased mitochondrial superoxide levels (**D**) as determined using the MitoSOX red mitochondrial superoxide indicator. Values are mean ± SEM; *N* = 6–12. *p < 0.05 *vs.* scrambled siRNA.

**Figure 3:**
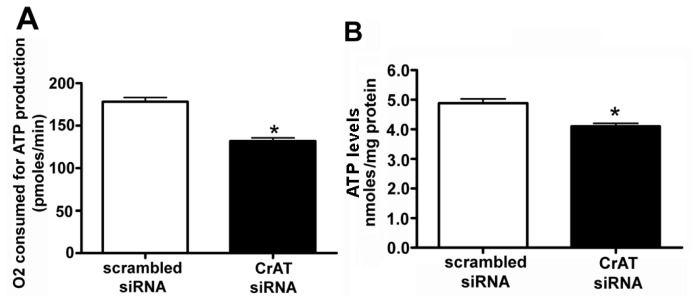
Decreased CrAT activity disrupts mitochondrial bioenergetics and ATP generation in pulmonary arterial endothelial cells. PAEC were transiently transfected with a scrambled siRNA or a specific CrAT siRNA for 48 h and the effect on mitochondrial respiration determined using the Seahorse XF24 analyzer. In the CrAT siRNA transfected cells there was a significant decrease in the (**A**) amount of oxygen consumed (OCR) for ATP production and (**B**) this corresponded with a reduction in cellular ATP levels. Values are mean ± SEM; *N* = 6. *p < 0.05 *vs.* scrambled siRNA.

SOD2 is known to be a major mitochondrial superoxide scavenger enzyme that protects the cells against oxidative stress. We found that suppressing CrAT activity led to a significant decrease in SOD2 mRNA (Figure 2A), SOD2 protein (Figure 2B) and SOD2 activity (Figure 2C). In turn we were able to show the decrease in SOD2 activity led to a significant increase in mitochondrial superoxide levels (Figure 2D) indicating that CrAT-regulated loss of SOD2 resulted in increased oxidative stress within the mitochondria. Further, our data indicate that CrAT gene silencing produces significant decrease both in O_2_ consumed for ATP production (Figure 3A) and cellular ATP levels (Figure 3B). Together these data indicate that the loss of carnitine homeostasis induced by suppressing CrAT activity, leads to the disruption of mitochondrial function.

### 2.3. Suppressing CrAT Expression Causes Nitrative Stress in Pulmonary Arterial Endothelial Cells

**Figure 4:**
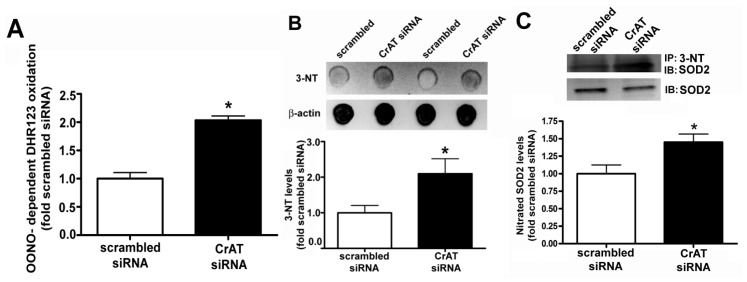
CrAT gene silencing causes nitrative stress in pulmonary arterial endothelial cells. PAEC were transiently transfected with a scrambled siRNA or a specific CrAT siRNA for 48 h and the effect on cellular peroxynitrite levels determined. There was an increase in peroxynitrite generation (**A**) as determined by DHR123 oxidation and a corresponding increase in total nitrated proteins; (**B**) as determined by Dot Blot analysis (**C**) This increase in peroxynitrite generation resulted in a significant increase of SOD2 nitration. Values are mean ± SEM; *N* = 6–11. *p < 0.05 *vs.* scrambled siRNA.

To determine if the increase in oxidative stress associated with mitochondrial dysfunction also resulted in increased nitrative stress, we next determined cellular peroxynitrite levels using the oxidation of DHR 123 to rhodamine 123 and protein nitration using an antibody specific for 3-NT residues. Our data indicate that silencing the CrAT gene increased both peroxynitrite (Figure 4A) and total protein nitration (Figure 4B). Further, despite less SOD2 present in CrAT siRNA transfected cells (Figure 2A,B), the increase in cellular peroxynitrite generation increased SOD2 nitration (Figure 4C).

### 2.4. CrAT Mediated Mitochondrial Dysfunction Disrupts eNOS/Hsp90 Interactions and Leads to eNOS Uncoupling

**Figure 5:**
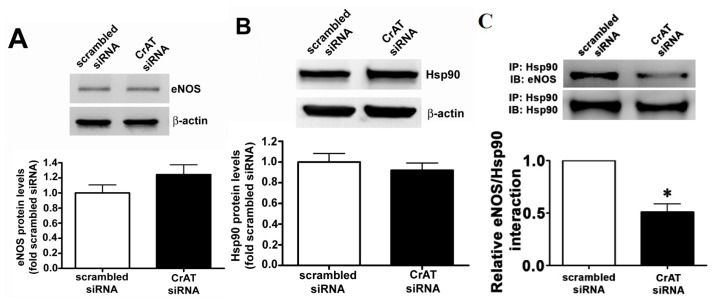
CrAT gene knockdown disrupts eNOS/Hsp90 interactions in pulmonary arterial endothelial cells. PAEC were transiently transfected with a scrambled siRNA or a specific CrAT siRNA for 48 h. Western blot analysis revealed that reducing CrAT expression did not alter total protein levels of (**A**) eNOS or (**B**) Hsp90 (**C**) However, immunoprecipitation (IP) analysis using a specific antiserum raised against Hsp90 followed by Western blot (IB) analysis with an anti-eNOS antibody revealed that there was a significant decrease in the association of eNOS with Hsp90 in CrAT siRNA transfected cells. The membrane was then reprobed for Hsp90 to normalize for immunoprecipitation efficiency. Values are mean ± SEM; *N* = 6. *p < 0.05 *vs.* scrambled siRNA.

**Figure 6:**
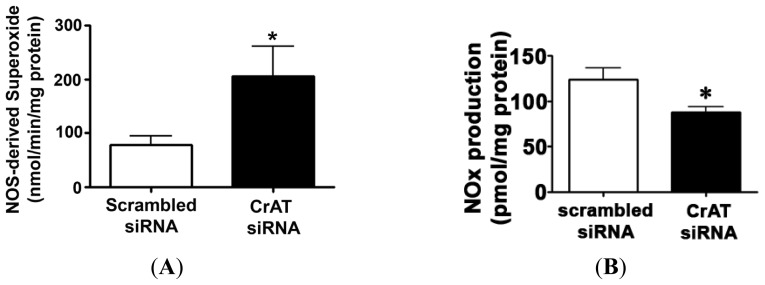
CrAT gene silencing attenuates shear stress induced NO signaling in pulmonary arterial endothelial cells. PAEC were transiently transfected with a scrambled siRNA or a specific CrAT siRNA for 48 h then acutely exposed to laminar shear stress (20 dyn/cm^2^, 15 min) in the presence or absence of the NOS inhibitor 2-ethyl-2-thiopseudourea (ETU; 100 μM, 30 min) and the effect on eNOS-derived superoxide generation determined by EPR (**A**) Decreasing CrAT activity significantly increased superoxide levels. The presence of ETU significantly inhibited the increase in superoxide levels in the CrAT siRNA transfected cells, indicating that it is eNOS-dependent (**B**) Conversely, the shear-mediated increase in NO_x_ was significantly decreased in CrAT siRNA transfected PAEC. Values are mean ± SEM; *N* = 6; * *p* < 0.05 *vs.* scrambled siRNA.

Hsp90 is an ATP dependent chaperone and the interaction of Hsp90 with eNOS has been shown to increase eNOS coupling and NO production [20]. Thus, we next examined the effect of the decrease in cellular ATP levels on eNOS/Hsp90 interactions. CrAT gene silencing did not alter total eNOS (Figure 5A) or Hsp90 (Figure 5B) protein levels in PAEC. However, immunoprecipitation analyses revealed that siRNA mediated CrAT knockdown significantly decreased the interaction of Hsp90 to eNOS (Figure 5C). Further, we found that this disruption in eNOS/Hsp90 interaction induced a significant increase in NOS-derived superoxide levels (Figure 6A) and a significant reduction in NO generation (Figure 6B) when PAEC were acutely exposed to fluid shear stress. Together these data indicate that the impaired mitochondrial function induced by decreased CrAT activity leads to uncoupling of eNOS and reduced NO signaling.

## 3. Discussion

Carnitine plays a vital role in cellular energy production. In the mitochondria, carnitine exists in a balance with acetyl-l-carnitine and acetyl-CoA that is involved in regulating mitochondrial activity and fat burning. Studies have shown that carnitine has a protective effect both on mitochondria and in whole cells by inhibiting free fatty acid induced mitochondrial membrane damage and/or its secondary effects [21–23]. Recent experimental and clinical studies have shown that mitochondrial dysfunction secondary to a disruption of carnitine homeostasis may play a role in decreased NO signaling and the development of endothelial dysfunction [19,24]. Carnitine is present in the organism as free carnitine (FC) or as acylcarnitines (AC, esterified form), which along with carnitine-dependent enzymes and plasma membrane transporters constitute the carnitine system. Adequate carnitine levels, as well as optimal activities of carnitine-dependent enzymes are required to maintain balanced carnitine homeostasis. The main function of l-carnitine is the transport of long-chain fatty acids from the cytosol to the mitochondrial matrix for β*-*oxidation and ATP production. l-carnitine however, also plays a key regulatory role in intermediary metabolism by modulating cellular acyl-CoA/CoA ratio. This function is mostly dependent on the freely reversible conversion of short-chain acyl-CoA and carnitine to free CoA and acylcarnitine by the intra-mitochondrial enzyme, CrAT. The acetyl-carnitine shuttle in which acetyl-CoA is reversibly converted to acetyl-carnitine by carnitine acetyl transferase (CrAT) enzymes is important for intracellular transport of acetyl units.

Coenzyme A is an obligate cofactor for many enzymes involved in intermediary metabolism. It remains compartmentalized in limited pools within the cell, mainly in the mitochondria, and is normally kept in homeostasis with carnitine. The reversible transfer of acyl groups from CoA to carnitine ensures the vital maintenance of free CoA pools within the mitochondria and prevents the accumulation of poorly metabolized short-chain acyl-CoA compounds, which are exported out of the mitochondria as carnitine esters. Therefore, the carnitine system is crucial for normal mitochondrial function, as the accumulation of acyl groups and the unavailability of free CoA result in a metabolic roadblock within the mitochondria, with subsequent impaired oxidative metabolism, increased mitochondrial ROS generation, and decreased energy production [25–27]. We recently identified a disruption in carnitine homeostasis in shunt lambs that correlated with mitochondrial dysfunction, oxidative stress, and impaired NO signaling [19]. These lambs showed high acylcarnitine: free carnitine ratio, reflecting an imbalance in mitochondrial acylCoA/CoA, as well as decreased carnitine-dependent enzymes (CPT1, CPT2 and CrAT) and a significant decrease in CrAT activity [19]. Despite compelling evidence that oxidative stress plays a causal role in the development of pulmonary vascular disease secondary to increased PBF [2], this study was the first to suggest a mitochondrial component linked to alterations in the carnitine system in its pathogenesis. Previously, lung mitochondrial dysfunction had only been reported in the pulmonary hypertension syndrome of fast-growing broilers syndrome, which was interestingly attenuated by antioxidant therapy with vitamin E [28]. Different mechanisms could explain the disrupted carnitine homeostasis in our lamb model of increased PBF as we have observed changes in CPT1, CPT2, and CrAT. However, the cell culture data presented here suggest decreasing CrAT activity is sufficient to induce mitochondrial dysfunction and NO signaling.

In the present study, we found that knockdown of CrAT using siRNA approach resulted in an increase in peroxynitrite and total nitrated proteins thus, suggesting higher nitrative stress in the endothelial cells. SODs play a critical role in inhibiting oxidative inactivation of NO, thereby preventing peroxynitrite formation. Both oxidative and nitrative stress within the mitochondria can cause mitochondrial dysfunction by damaging mitochondrial proteins and thereby altering electrochemical gradient. A study undertaken to examine the oxidation-induced apoptosis in cultured mouse retinal pigment epithelial cells, has shown that the deficiency of SOD2 resulted in greater disruption of the membrane potential, whereas over-expression of the enzyme protected against mitochondrial membrane damage. It was also reported that the extent of the mitochondrial damage was related to the level of SOD2 [29]. We found that CrAT gene silencing decreased SOD2 expression and increased SOD2 nitration. Together these changes produce a decrease in SOD2 activity. This link between carnitine homeostasis, SOD2, and mitochondrial dysfunction may play an important role in the development of pulmonary hypertension as we have shown early derangements in SOD2 in our lamb model of pulmonary hypertension [30] while a recent study found that SOD2 expression and activity was decreased in PAEC isolated from patients with idiopathic pulmonary arterial hypertension (IPAH) [31]. Similarly, both carnitine metabolism and fatty acid oxidation are significantly depressed in human pulmonary vascular endothelial cells containing a bone morphogenetic protein receptor type 2 (BMPR2) mutation [32] again suggesting a link between carnitine metabolism and mitochondrial dysfunction in pulmonary hypertension.

It has been previously shown that SOD2, which plays a critical role in cellular defense against oxidative stress by decomposing superoxide within mitochondria, is nitrated and inactivated under pathological conditions [33]. Therefore, our data suggest that CrAT might be regulating SOD2 both at transcriptional (protein) and post-translational (nitration) levels. Indeed, several previously published studies support this finding. For example, l-carnitine has been shown to enhance SOD activity in a number of cell types [34–36] suggesting that there is a link between carnitine homeostasis and the regulation of SOD gene expression. Similar to our results, a previous study has also shown that a reduction in SOD2 levels results in increased superoxide and peroxynitrite concentrations and decreased NO concentration in the vessel wall [37]. It has also been reported that the manganese ion in SOD2 enzyme plays an important role in the decomposition kinetics of peroxynitrite and in peroxynitrite-dependent nitration of self and remote tyrosine residues [38].

In this study we also found a significant decrease in ATP levels in CrAT siRNA transfected cells which corresponded to a reduction in oxygen consumption rate (OCR) related to ATP synthase. Previous studies have shown the importance of ATP in pulmonary endothelial function, at least in part, through its ability to stimulate NO release via the activation of eNOS [39]. Endothelial NOS activity is tightly controlled through multiple mechanisms that include phosphorylation and protein-protein interactions [40]. Hsp90, a member of a molecular chaperone family, is among the proteins that increase eNOS activity by facilitating the displacement of caveolin-1 from eNOS, in a process that is ATP dependent [41]. Therefore, it is plausible that the reduction in ATP levels associated with reduced CrAT expression decreases Hsp90/eNOS interactions and attenuates NO production [19]. Further, it is suggestive that l-carnitine supplementation could improve endothelial function. A number of studies have evaluated l-carnitine as a therapeutic tool in conditions characterized by mitochondrial dysfunction and oxidative stress. In addition to reducing the toxicity resulting from excess acyl-CoA, exogenous l-carnitine has been shown to have antioxidant and anti-apoptotic properties [11,42,43]. The mechanisms by which l-carnitine protect cells against ROS is not completely clear, but may include direct free radical scavenging and inhibition and/or repair of peroxidized biomolecules [43,44]. Exogenous l-carnitine has shown a beneficial effect in both animal and human studies in conditions as diverse as Alzheimer disease, hypoxic-ischemic brain injury, diabetes, aging, chronic renal failure, atherosclerosis or ischemic heart disease [21,43–50]. In each case oxidative stress was reduced and mitochondrial performance was enhanced. l-carnitine supplementation in systemic hypertensive rats has also been previously shown to enhance NO production, while attenuating oxidative stress and endothelial dysfunction [51,52].

## 4. Methods

### 4.1. Culture of Pulmonary Arterial Endothelial Cells

Primary cultures of ovine pulmonary arterial endothelial cells (PAEC) were isolated as described previously [53]. All cultures were maintained in DMEM supplemented with 10% fetal calf serum (Hyclone, Logan, UT, USA) antibiotics/antimycotic (500 IU Penicillin, 500 μg/mL Streptomycin, 1.25 μg/mL Amphotericin B; MediaTech, Herndon, VA, USA) at 37 °C in a humidified atmosphere with 5% CO_2_ and 95% air. Cells between passages 3 and 10 were used for all experiments.

### 4.2. Targeted Silencing of Carnitine Acetyl Transferase by Small-Interfering RNA

PAEC were grown in 6-well plates to ~60% confluence and transfected with optimized concentrations of a custom made small interfering RNA (siRNA) specific for ovine CrAT (s100014889, Santa Cruz Biotechnology, Santa Cruz, CA, USA) or as a control, a scrambled siRNA (sc-37007, Santa Cruz Biotechnology, Santa Cruz, CA, USA) with no known homology to any sequences from mouse, rat, or human RNA. Transfections were performed using the HiPerfect transfection reagent (cat # 301705, Qiagen, Valencia, CA, USA) and 25 nM of the appropriate siRNA. Whole cell lysates were prepared after 48 h of transfection, and CrAT knockdown confirmed using Western blot analysis.

### 4.3. Sample Purification and Measurement of Carnitine Metabolites

For free carnitine determination, 100 μL cell lysates, 300 μL of water, and 100 μL of an internal standard (Sigma ST 1093) were mixed. For total carnitine determinations, 100 μL cell lysates were hydrolyzed with 0.3 M KOH, heated at 45 °C, and pH neutralized using 0.8 M perchloric acid; the final volume was made to 400 μL and 100 μL of an internal standard was added. The total volume of each reaction mixture should be 500 μL. All samples were purified using solid-phase extraction columns (SAX, 100 mg/mL; Varian, Harbor City, CA, USA), derivatized using aminoanthracene in the presence of a catalyst; 1-[3-(dimethylamino)propyl]-3-ethylcarbodiimide hydrochloride (EDCI), and kept at 30 °C for an hour. Separation was carried out using an isocratic elution in 0.1 M Tris-acetate buffer (pH 3.5): acetonitrile (68:32, *v*/*v*) at a flow rate of 0.9 mL/min as described previously [19]. Detection of carnitines was performed using a Shimadzu UFLC system with a 5 μm Omnispher C18 column (250 × 4.6 mm OD) and equipped with an RF-10AXL fluorescence detector (Shimadzu USA Manufacturing Corporation, Canby, OR, USA). Total and free carnitine levels were quantified by fluorescence detection at 248 nm (excitation) and 418 nm (emission). The acylcarnitines were calculated by subtracting the free carnitine values from the total carnitine values for all the samples.

### 4.4. Measurement of Peroxynitrite Levels

The formation of peroxynitrite was determined by the oxidation of dihydrorhodamine (DHR) 123 to rhodamine 123, as described previously [54]. Cultured PAEC were transfected with either scrambled or CrAT siRNA for 48 h. The cells were then treated with PEG-Catalase (100 U, 30 min) to reduce H_2_O_2_ dependent DHR 123 oxidation. Five micromole per litre DHR 123 was added to the cells in phenol red-free media and the fluorescence of rhodamine 123 was measured at excitation 485 nm and emission 545 nm after 30 min of incubation using a Fluoroskan Ascent Microplate Fluorometer. The fluorescent values were normalized to the protein levels in the samples.

### 4.5. Measurement of 3-NT Levels

The total nitrated protein levels were measured in the PAEC transfected with either scrambled or CrAT siRNA via a dot blot procedure. Briefly, 50 μg protein lysate was applied to a nitrocellulose membrane pre-soaked with Tris-buffered saline (TBS). After the protein samples were completely transferred, the membrane was blocked in 5% fat-free milk for 1 h, washed with TBS, and incubated with mouse anti-3-nitrotyrosine (1:100, Calbiochem) antibody overnight. Finally, the membrane was incubated with goat anti-mouse IgG for 2 h. The reactive dots were visualized using chemiluminescence (Pierce Laboratories, Rockford, IL, USA) on a Kodak 440 CF image station (New Haven, CT, USA). The band intensity was quantified using Kodak 1D image processing software. Protein loading was normalized by re-probing with mouse anti β-actin antibody.

### 4.6. Real Time Quantitative (q) RT-PCR for mRNA Levels

Quantitative RT–PCR using SYBR green I dye for specific detection of double-stranded DNA was employed to determine SOD2 mRNA levels in scrambled siRNA and CrAT siRNA transfected (48 h) PAEC. Briefly, total RNA was extracted using the RNeasy kit (Qiagen), and 1 μg total RNA was reverse-transcribed using QuantiTect Reverse Transcription Kit (Qiagen) in a total volume of 20 μL Primers for SOD2 and β-actin were designed by IDT (Coralville, IA, USA). The sequences were SOD2 Forward, 5′-GTTGGCTCGGCTTCAATAAG-3′, Reverse, 5′-AATCGGGCCTGACATTTTTA-3′; β-actin Forward, 5′-GGGAAATCGTGCGTGACATTAAG-3′, Reverse, 5′-TGTGTTGGCGTAAGGT CTTTG-3′. Real-time PCR and melting curve analyses were carried out using an Mx4000 Multiplex Quantitative PCR System (Stratagene), using 2 μL of RT product, 12.5 μL of QuantiTect SYBR Green PCR Master Mix (Qiagen) and primers (400 nM) in a total volume of 25 μL. The following thermocycling conditions were employed: 95 °C for 10 min, followed by 95 °C for 30 s, 55 °C for 60 s and 72 °C 30 s for 40 cycles. There were 2^−ΔΔCT^ values chosen to reflect the number of mRNA molecules using β-actin (housekeeping gene) as an internal control.

### 4.7. Western Blot Analyses

Protein extracts were prepared by homogenizing CrAT siRNA transfected PAEC in Triton lysis buffer (50 mM Tris-HCL, pH 7.6, 0.5% Triton-X100, 20% glycerol) containing a protease inhibitor cocktail. Extracts were then clarified by centrifugation (15,000 rpm for 10 min at 4 °C). Supernatant fractions were then assayed for protein concentration using the Pierce BCA protein assay kit (ThermoScientific, Rockford, IL, USA), and Western blot analysis was performed as previously described [55–57]. Briefly, protein extracts (25–50 μg) were separated on Long-Life 4%–20% Tris-SDS-Hepes gels (Frenchs Forest, Australia). All gels were electrophoretically transferred to Immuno-Blot™ PVDF membrane (Bio-Rad Laboratories, Hercules, CA, USA). The membranes were blocked with 5% nonfat dry milk in Tris-buffered saline containing 0.1% Tween 20 (TBST). After blocking, the membranes were probed at room temperature with antibodies to CrAT (Proteintech, Chicago IL, USA); SOD2 (Upstate, Lake Placid, NY, USA); eNOS or Hsp90 (BD Transduction, San Jose, CA, USA); washed with TBS containing 0.1% Tween, and then incubated with an appropriate IgG conjugated to horseradish peroxidase. Protein bands were then visualized with chemiluminescence (SuperSignal^®^ West Femto Substrate Kit, Pierce Laboratories, Rockford, IL, USA) on a Kodak 440 CF Image Station (Kodak, Rochester, NY, USA). Band intensity was quantified using Kodak 1D image processing software. All captured and analyzed images were determined to be in the dynamic range of the system. To normalize for protein loading, blots were re-probed with the housekeeping protein, β-actin.

### 4.8. Measurement of SOD2 Activity

SOD2 activity was measured in whole cell homogenates from scrambled and CrAT siRNA transfected PAEC using a SOD activity kit (Enzo Life Sciences, Farmingdale, NY, USA) according to the manufacturer’s instructions. Absorbance was read at 450 nm and SOD2 activity presented as units per microgramme protein.

### 4.9. Immunoprecipitation Analysis

To determine the levels of nitrated SOD2 and eNOS/Hsp90 interactions after CrAT siRNA transfections, PAEC were homogenized in immunoprecipitation buffer 25 mM HEPES, pH 7.5, 150 mM NaCl, 1% Nonidet *P*-40, 10 mM MgCl_2_, 1 mM EDTA, and 2% glycerol supplemented with protease inhibitor cocktail (Pierce Laboratories, Rockford, IL, USA). Cell homogenates (500 μg of protein) were precipitated with either 3-nitrotyrosine or rabbit antibody against Hsp90 in 0.5 mL final volume at 4 °C overnight. Protein G plus/protein A agarose (40 μL; Calbiochem) was added and rotated at 4 °C for an additional 2 h. The precipitated protein was washed three times in 2× volume of immunoprecipitation buffer; the pellet was re-suspended in Laemmli buffer (20 μL), boiled, and separated on a 4%–20% SDS-PAGE gel (LongLife). eNOS protein levels were then detected using Western blot analysis as described above. The efficiency of immunoprecipitation was normalized by reprobing the membranes with anti-Hsp90 antibody. The nitrated SOD2 blot was normalized by running the same samples in a separate gel for total SOD2 protein levels.

### 4.10. Determination of Mitochondrial Superoxide

MitoSOX™ Red mitochondrial superoxide indicator (Molecular Probes), a fluorogenic dye for selective detection of superoxide in the mitochondria of live cells was used. The MitoSOX Red reagent is live-cell permeant and is rapidly and selectively targeted to the mitochondria. Once in the mitochondria, MitoSOX Red reagent is oxidized by superoxide and exhibits bright red fluorescence upon binding to nucleic acids. After siRNA mediated CrAT silencing, cells were washed with fresh media, and then incubated in media containing MitoSOX Red (5 μM), for 30 min at 37 °C in dark conditions. Cells were washed with fresh serum-free media and imaged using fluorescence microscopy at an excitation of 510 nm and an emission at 580 nm. A PC-based imaging system consisting of the following components was used for the fluorescent analyses: an Olympus IX51 microscope equipped with a CCD camera (Hamamatsu Photonics, Bridgewater, NJ, USA) was used for acquisition of fluorescent images. The average fluorescent intensities (to correct for differences in cell number) were quantified using ImagePro Plus version 5.0 imaging software (Media Cybernetics, Rockville, MD, USA) as previously published [58].

### 4.11. Mitochondrial Bioenergetic Analysis in Transfected Cells

The XF24 Analyzer (Seahorse Biosciences, North Billerica, MA, USA) was used to measure bioenergetics function in scrambled and CrAT siRNA transfected endothelial cells. In preliminary studies, the optimum number of cells/well was determined as 75,000/0.32 cm^2^. This cell number allows the appropriate detection of changes in oxygen consumption rate (OCR). Then the electron transport chain uncouplers and inhibitors such as Oligomycin, FCCP (carbonyl cyanide 4-(trifluoromethoxy phenylhydrazone) and rotenone + antimycin A were injected sequentially through ports of the Seahorse Flux Pak cartridges to reach final concentrations of 1 uM each. Using these agents, we determined the amount of oxygen consumption linked to ATP production and the data presented as picomoles per minute.

### 4.12. Determination of Cellular ATP Levels

ATP levels were estimated using the firefly luciferin-luciferase method utilizing a commercially available kit (Invitrogen) as previously published [19]. ATP is consumed and light is emitted when firefly luciferase catalyzes the oxidation of luciferin. The amount of light emitted during the reaction is proportional to the availability of ATP. Luminescence was determined using a Fluoroscan Ascent FL plate luminometer (ThermoFisher Scientific, Waltham, MA, USA). ATP levels were presented as nanomoles per milligram of protein.

### 4.13. Shear Stress

PAEC were exposed to laminar shear stress using a cone-plate viscometer that accepts six-well tissue culture plates, as described previously [59]. This method achieves laminar flow rates that represent physiological levels of laminar shear stress in the major human arteries, which is in the range of 5–20 dyn/cm^2^ [60]. Cells were acutely exposed to shear stress (20 dyn/cm^2^, 15 min) and both NOS-derived superoxide and NO*_x_* levels determined.

### 4.14. Measurement of NOS-Derived Superoxide Levels in PAEC

This was estimated by electron paramagnetic resonance (EPR) assay using the spin-trap compound 1-hydroxy-3-methoxycarbonyl-2,2,5,5-tetramethylpyrrolidine HCl (CMH, Enzo Life Sciences, Inc., Farmingdale, NY, USA) as described previously [20,61]. Superoxide produced in PAEC was trapped by incubating cells with 20 μL of CMH stock solution (20 mg/mL) for 1 h. The cells were then trypsinized and centrifuged at 500*g* for 5 min. The cell pellet was suspended in 35 μL DPBS and loaded into a capillary tube which was then analyzed with a MiniScope MS200 EPR machine (Magnettech, Berlin, Germany). NOS-derived superoxide was measured by pre-incubating lysate with 100 μM ethylisothiourea (ETU, Sigma-Aldrich, St. Louis, MO, USA) for 30 min followed by incubation with CMH. EPR spectra were analyzed using ANALYSIS v.2.02 software (Magnettech: Berlin, Germany, 2005). Differences between levels of samples incubated in the presence and absence of ETU were used to determine NOS-dependent superoxide generation. Superoxide levels were reported as nmols superoxide/min/mg protein.

### 4.15. Determination of Cellular NO_x_ Levels

NO and its metabolites were determined in PAEC homogenates to quantify bioavailable NO. In solution, NO reacts with molecular oxygen to form nitrite, and with oxyhaemoglobin and superoxide anion to form nitrate. Nitrite and nitrate are reduced using vanadium (III) and hydrochloric acid at 90 °C. NO is purged from solution resulting in a peak of NO for subsequent detection by chemiluminescence (NOA 280, Sievers Intruments Inc., Boulder, CO, USA), as we have previously described [62,63]. The sensitivity is 1 × 10^−12^ moles, with a concentration range of 1 × 10^−9^ to 1 × 10^−3^ molar of nitrate.

### 4.16. Statistical Analysis

Statistical analysis was performed using GraphPad Prism version 4.01 for Windows (GraphPad Software: San Diego, CA, USA). The mean ± SEM was calculated for all samples and significance was determined by the unpaired *t*-test. A value of *p* < 0.05 was considered significant.

## 5. Conclusion

Our results indicate that CrAT is an important enzyme that is not only involved in optimizing mitochondrial function, but is also involved in maintaining SOD2 expression and decreasing mitochondrial oxidative stress. Further, by maintaining cellular ATP levels, both Hsp90 activity and NO signaling are preserved. We suggest that chronic l-carnitine therapy may improve and/or attenuate the decline in endothelial function noted in children with CHD and increased PBF, and thus may have important clinical implications that warrant further investigation.
